# Urinary Properdin and sC5b-9 Are Independently Associated With Increased Risk for Graft Failure in Renal Transplant Recipients

**DOI:** 10.3389/fimmu.2019.02511

**Published:** 2019-10-24

**Authors:** Rosa G. M. Lammerts, Michele F. Eisenga, Mohammed Alyami, Mohamed R. Daha, Marc A. Seelen, Robert A. Pol, Jacob van den Born, Jan-Stephan Sanders, Stephan J. L. Bakker, Stefan P. Berger

**Affiliations:** ^1^Division of Nephrology, Department of Internal Medicine, University Medical Center Groningen, University of Groningen, Groningen, Netherlands; ^2^Department of Surgery, University Medical Center Groningen, University of Groningen, Groningen, Netherlands

**Keywords:** transplantation, chronic renal failure, properdin, C5b-9, complement activation

## Abstract

The pathophysiology of late kidney-allograft failure remains complex and poorly understood. Activation of filtered or locally produced complement may contribute to the progression of renal failure through tubular C5b-9 formation. This study aimed to determine urinary properdin and sC5b-9 excretion and assess their association with long-term outcome in renal transplant recipients (RTR).

**Methods:** We measured urinary properdin and soluble C5b-9 in a well-defined cross-sectional cohort of RTR. Urinary specimens were taken from a morning urine portion, and properdin and sC5b-9 were measured using an enzyme-linked-immunosorbent assay (ELISA). Cox proportional hazard regression analyses were used to investigate prospective associations with death-censored graft failure.

**Results:** We included 639 stable RTR at a median [interquartile range] 5.3 (1.8–12.2) years after transplantation. Urinary properdin and sC5b-9 excretion were detectable in 161 (27%) and 102 (17%) RTR, respectively, with a median properdin level of 27.6 (8.6–68.1) ng/mL and a median sC5b-9 level of 5.1 (2.8–12.8) ng/mL. In multivariable-adjusted Cox regression analyses, including adjustment for proteinuria, urinary properdin (HR, 1.12; 95% CI 1.02–1.28; *P* = 0.008) and sC5b-9 excretion (HR, 1.34; 95% CI 1.10–1.63; *P* = 0.003) were associated with an increased risk of graft failure. If both urinary properdin and sC5b-9 were detectable, the risk of graft failure was further increased (HR, 3.12; 95% CI 1.69–5.77; *P* < 0.001).

**Conclusions:** Our findings point toward a potential role for urinary complement activation in the pathogenesis of chronic allograft failure. Urinary properdin and sC5b-9 might be useful biomarkers for complement activation and chronic kidney allograft deterioration, suggesting a potential role for an alternative pathway blockade in RTR.

## Introduction

Despite improvements in immunosuppressive therapy over the last decades, chronic and irreversible deterioration of a transplanted kidney graft remains a major problem and is responsible for disappointing outcomes in long-term graft survival (1). Even though registry data can be used to define risk factors, chronic allograft failure pathophysiology remains complex and poorly understood, due to difficulty in distinguishing the contribution of several immunological and non-immunological factors (2). Interstitial fibrosis/tubular atrophy (IFTA), presents itself as renal allograft dysfunction (occurring at least 3 months post-transplant) in the absence of active acute rejection, drug toxicity, or other diseases. Due to its multiple possible causes and complex etiology, classification of IFTA is still an ongoing process (3, 4). The clinical diagnosis is usually suggested by gradual deterioration of allograft function, manifested by a slowly rising serum creatinine concentration, worsening hypertension, and increasing proteinuria. Proteinuria is known to be a progression marker and a predictor for renal failure (5, 6). It is thought that proteinuria contributes to the progression of renal failure by various mechanisms. One of these mechanisms is suggested to be leakage of albumin-bound lipids across the damaged glomerular filtration barrier, leading to lipoapoptosis after reabsorption by the downstream proximal tubule (7, 8). Alongside this, activation of filtered or locally produced complement may be harmful to renal tubular cells and contribute to the progression of renal failure by initiating interstitial fibrosis (9, 10). Complement activation leads to the formation of C5b-9 (11), which can be used as a clinical indicator of complement activation in native kidney diseases (12, 13).

Renal proximal tubular cells are known to activate complement via the alternative pathway (AP) (14). Gaarkeuken et al. showed that complement activation on tubular cells is mediated by properdin binding on the tubular brush border (15). Our group identified tubular heparan sulfate as the docking platform for properdin and the consequent AP activation on tubular cells (9). In proteinuric patients, urinary properdin excretion is associated with intrarenal complement activation and poor renal function (16, 17).

Although it has been established that there is a strong relationship between proteinuria, tubulo-interstitial injury and a poor prognosis in kidney disease, to our knowledge no studies have examined the role of urinary complement activation products in kidney transplantation outcomes.

We hypothesized that the AP regulator properdin and the terminal complement complex sC5b-9 play an important role in graft failure and could serve as early biomarkers for late graft failure. Hence, the aim of the present study is to investigate the role of properdin and sC5b9 in renal transplant recipients (RTR) in relation to the development of graft failure over time.

## Methods

### Study Population

The study population consisted of a well-characterized and previously described cohort of 707 RTR (18). In short, this cohort comprised RTR (aged ≥ 18 years) who visited the outpatient clinic of the University Medical Center Groningen (UMCG), Groningen, The Netherlands, between November 2008 and June 2011, and who had a functional graft for at least 1 year after transplantation. All patients provided written informed consent. Urinary morning samples were collected at inclusion in the study and immediately placed on ice. The samples were centrifuged at 4°C at 4,000 RPM for 15 min to remove components and debris, and the supernatants were stored at −80°C. They were not subjected to freeze/thaw cycles before analysis. There were 639 patients eligible for analysis after we excluded 67 patients with missing urinary samples which precluded the measurement of urinary properdin and sC5b-9 levels. Death-censored graft failure was defined as return to dialysis or re-transplantation. Kidney function was assessed by estimating glomerular filtration rate (eGFR) by applying the Chronic Kidney Disease Epidemiology Collaboration equation (19). Protein excretion of ≥0.5 g per day was defined as proteinuria. The study was approved by the UMCG institutional review board (METc 2008/186), adheres to the Declarations of Helsinki and Istanbul and has NCT02811835 as ClinicalTrials.gov identifier.

### Quantification of Urinary Properdin

Urinary properdin levels were assessed by a previously described sandwich enzyme-linked-immunosorbent assay (ELISA) (9, 17), with a detection limit of 1.2 ng/mL, a plasma intra-variation of <17% and an inter-variation of <20%. In brief, 96-well ELISA plates (NUNC MaxiSorp^TM^, Sigma-Aldrich, Saint Louis, MO, USA) were coated overnight at 4°C with monoclonal anti-human properdin (Hycult HM2282, Uden, the Netherlands). Urinary samples were diluted 5 times in DPBS with 0.1% Tween and bovine serum albumin (PTB) and incubated for 1 h at 37°C, followed by secondary antibody; polyclonal rabbit anti-human properdin-biotin (kindly provided by M. R. Daha, Leiden, The Netherlands) and detection with Streptavidin-HRP (Dako P0397, Glostrup, Denmark). Enzyme activity was detected using 2,2′azino-bis (3-ethylbenzo-thiazoline-6-sulphonic acid) (A1888, Sigma-Aldrich, Saint Louis, MO, USA). The optical density was measured at 415 nm using a microplate ELISA reader (Benchmark Plus, Bio-Rad, Veenendaal, The Netherlands). A standard curve was prepared using a serial dilution of zymosan activated serum in PTB with a known concentration of properdin. A reference sample, diluted in PTB with a known concentration of properdin was included as positive control. Potential background signal was assessed and corrected for, with PTB functioning as blank.

### Quantification of Urinary Soluble C5b-9

Urinary sC5b-9 levels were assessed by a previously standardized and validated sandwich ELISA (17, 20), with a detection limit of 2.1 ng/mL, a plasma intra-variation of <13% and an inter-variation of <19%. In brief, 96-well ELISA plates (NUNC MaxiSorp^TM^, Sigma-Aldrich) were coated overnight at 4°C with monoclonal mouse anti-human C5b-9 (Dako M0777). Urinary samples were diluted 1.25 times and incubated for 1 h at 37°C. Secondary antibody polyclonal goat anti-human C5 (Quidel Ca92121, San Diego, CA, USA), followed by tertiary antibody polyclonal mouse anti-goat IgG HRP (Jackson 205-035-108) were added. Enzyme activity was detected using 3,3′,5,5′-tetramethylbenzidine. The optical density was measured at 450 nm using a microplate ELISA reader. The standard curve, reference sample, and the assessment of a potential background signal was prepared in the same way as the properdin ELISA, with a known concentration of sC5b-9.

### Statistical Analyses

Data were analyzed using IBM SPSS software, version 23.0 (SPSS Inc., Chicago, IL, USA) and R version 3.2.3 (Vienna, Austria). Data are expressed as mean ± SD for normally distributed variables and as median [25th−75th interquartile range (IQR)] for variables with a skewed distribution. Categorical data are expressed as number (percentage). Under normal conditions complement factors are not present in the urine. Therefore, we defined urinary properdin and sC5b9 as a negative test when undetectable in the urine and as a positive test when detectable.

We evaluated between-group differences at baseline, comparing RTRs with vs. without detectable properdin and sC5b-9 using Student *t*-test, Mann-Whitney *U*-test, or Chi square test, as appropriate. To visualize the association between urinary properdin and urinary sC5b-9 excretion, we generated a restricted cubic spline plot based on linear regression analyses. Knots were placed on the 10th, 50th, and 90th percentile of ln properdin. To visualize the association between urinary properdin and urinary sC5b-9 excretion with proteinuria, we generated restricted cubic spline plots based on linear regression analyses, with knots placed on the 10th, 50th, and 90th percentile of ln proteinuria. Further, Kaplan Meier curves were used to depict the effect of the presence of urinary properdin and/or sC5b-9 on graft failure and all-cause mortality. Differences in survival rates were tested using the Cox-Mantel log-rank test. To study the prospective association with death-censored graft failure and all-cause mortality, we used Cox proportional hazards regression analysis. Prior to analyses, we first adjusted for statistically significant different parameters at baseline and for other known predictors of graft failure like HLA mismatches. First, death-censored graft failure was adjusted for age, sex, primary renal disease, time since transplantation at inclusion, eGFR, HLA mismatches, and donor type (model 1). Additionally, adjustment was made for high sensitive-CRP (hs-CRP) (model 2); further adjustment for systolic blood pressure, and smoking (model 3); and final adjustment for proteinuria (model 4). Due to skewed distribution, hs-CRP, properdin, and sC5b-9 were natural log-transformed. To determine the optimal cut off value of urinary properdin and sC5b-9 for prediction of graft failure in RTR, the Youden index was used. Finally, we performed mediation analyses to assess whether sC5b-9 was a mediator in the association between properdin and graft failure. For this purpose, we used the method as stated by Preacher and Hayes, which is based on logistic regression (21, 22). These analyses allow for testing significance and magnitude of mediation. For all analyses, a two-sided *P* < 0.05 was considered significant.

## Results

### Baseline Characteristics

We included 639 RTR (age 53 ± 13 years; 58% males at 5.3 (1.8–12.2) years after transplantation). Mean eGFR was 52.2 ± 20.1 ml/min/1.73 m^2^, and urinary properdin excretion was detectable in 161 (27%) RTR with a median [interquartile range] properdin level of 27.6 (8.7–68.1) ng/mL. Urinary sC5b-9 excretion was detectable in 102 (17%) RTR with median sC5b-9 levels of 5.1 (2.8–12.8) ng/mL.

RTR with detectable urinary properdin were more frequently females (*P* < 0.001), had significantly higher: body surface area (m^2^) (*P* = 0.004), creatinine (*P* = 0.003), hs-CRP (*P* < 0.001), frequency of proteinuria (≥0.5 g/24 h) (*P* < 0.001), and received a deceased—donor kidney transplant (*P* = 0.02). RTR with detectable urinary sC5b-9 were more frequently males (*P* = 0.01), had higher levels of creatinine (*P* < 0.001), a higher frequency of proteinuria (*P* < 0.001), and a deceased-donor kidney transplant (*P* = 0.02). An inverse association between eGFR and detectable properdin (*P* < 0.001) and sC5b-9 levels (*P* < 0.001) was detected at baseline. No significant differences were found at baseline in HLA mismatches, primary renal disease, history of delayed graft function, and rejection between patients with and without detectable urinary properdin or sC5b-9.

**Figure 1:**
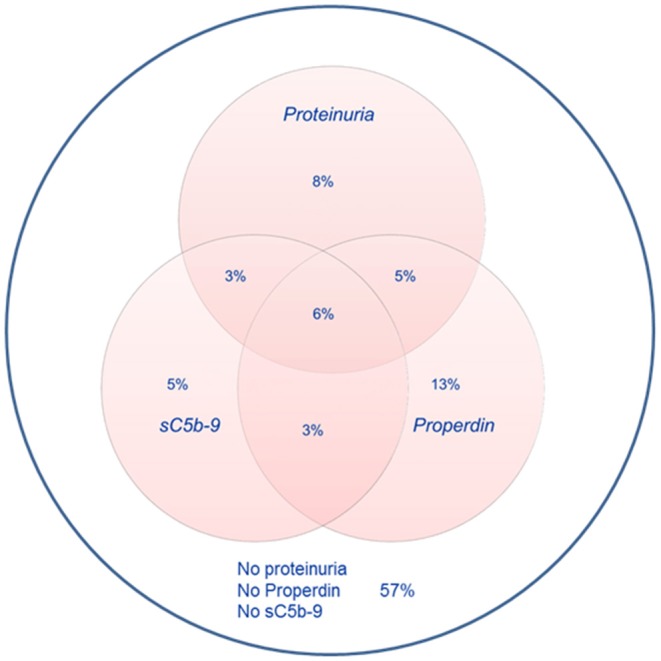
Prevalences of urinary properdin, urinary sC5b-9, and proteinuria.

**Figure 2:**
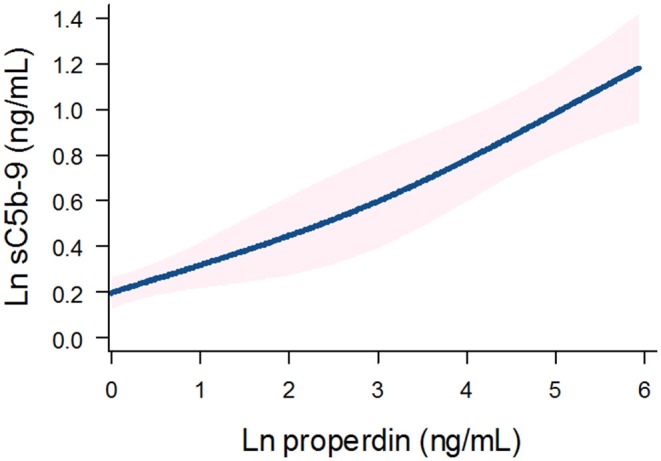
Association between urinary properdin and urinary sC5b-9 excretion in the RTR. A restricted cubic spline is generated based on linear regression analyses. Knots are placed on 10th, 50th, and 90th percentile of ln properdin. Blue line represents the coefficient, and pink band represents the 95% confidence interval.

Detectable urinary properdin excretion was present in 11 and 16% of RTR with and without proteinuria, respectively. Detectable urinary sC5b-9 excretion was present in 9 and 8% of RTR with and without proteinuria, respectively (Figure 1). Urinary properdin was significantly associated with urinary sC5b-9 excretion in RTR in whom both complement products were detectable (β = 0.25; *P* < 0.001) (Figure 2). Urinary properdin and urinary sC5b-9 excretion were both significantly associated with proteinuria (β = 0.26; *P* < 0.001 and β = 0.36; *P* < 0.001, respectively) (Supplementary Figures 1, 2). Further demographics and clinical characteristics dichotomized into detectable or undetectable urinary properdin and sC5b-9 are specified in Table 1.

**Table 1:** Baseline characteristics according to detectable urinary properdin urinary sC5b-9 levels.

**Variables**	**Urinary properdin**		***P*-value**	**Urinary sC5b-9**		***P*-value**
	**Not detectable (*n* = 478)**	**Detectable (*n* = 161)**		**Not detectable (*n* = 537)**	**Detectable (*n* = 102)**	
**RECIPIENT**						
Age (years)	53 ± 13	53 ± 13	0.96	53 ± 13	53 ± 13	0.88
Male sex (*n*, %)	305 (64)	66 (41)	<0.001	298 (56)	70 (69)	0.01
Body mass index, kg/m^2^	26.5 ± 4.2	26.7 ± 5.0	0.65	26.6 ± 4.7	26.3 ± 4.9	0.44
Body surface area (m^2^)	1.96 ± 0.21	1.90 ± 0.22	0.004	1.95 ± 0.21	1.94 ± 0.22	0.95
Alcohol use (*n*, %)	387 (82)	124 (77)	0.28	441 (82)	74 (73)	0.69
Current smoking (*n*, %)	55 (12)	20 (12)	0.72	58 (11)	17 (17)	0.06
Primary renal disease			0.34			0.95
Primary glomerular disease (*n*, %)	143 (30)	36 (22)		156 (29)	25 (25)	
Glomerulonephritis (*n*, %)	43 (9)	11 (7)		43 (8)	11 (11)	
Tubulo-interstitial disease (*n*, %)	48 (10)	25 (16)		63 (12)	11 (11)	
Polycystic renal disease (*n*, %)	95 (20)	36 (22)		109 (20)	22 (22)	
Dysplasia and hypoplasia (*n*, %)	19 (4)	6 (4)		22 (4)	4 (4)	
Renovascular disease (*n*, %)	29 (6)	8 (5)		30 (6)	7 (7)	
Diabetic nephropathy (*n*, %)	23 (5)	8 (5)		27 (5)	4 (4)	
Other or unknown cause (*n*, %)	78 (16)	31 (19)		87 (16)	18 (18)	
History of CV-disease (*n*, %)	58 (12)	23 (14)	0.31	71 (13)	10 (10)	0.65
Time since transplantation (years)^*^	5.3 (1.7–12.0)	6.1 (2.1–12.6)	0.39	5.1 (1.9–11.6)	7.1 (1.7–15.0)	0.07
Delayed graft function (*n*, %)	31 (7)	15 (9)	0.27	36 (7)	10 (10)	0.24
Rejection (*n*, %)	130 (27)	45 (28)	0.82	143 (27)	32 (31)	0.33
Diabetes mellitus (*n*, %)	109 (23)	38 (24)	0.75	124 (23)	23 (23)	0.84
Systolic blood pressure (mmHg)	136 ± 17	135 ± 18	0.84	135 ± 17	139 ± 19	0.05
Diastolic blood pressure (mmHg)	82 ± 11	82 ± 11	0.64	82 ± 11	85 ± 11	0.02
**LABORATORY MEASUREMENTS**						
sC5b-9 (ng/mL)	0 (0–0)	0 (0–3.8)	<0.001	0 (0–0)	5.1 (2.8–12.8)	
Properdin (ng/mL)	0 (0–0)	27.6 (8.7–68.1)		0 (0–0)	0 (0–32.4)	<0.001
Hemoglobin (mmol/L)	8.3 ± 1.1	7.9 ± 1.0	<0.001	8.2 ± 1.1	8.1 ± 1.2	0.31
Total cholesterol (mmol/L)	5.1 ± 1.1	5.2 ± 1.1	0.60	5.1 ± 1.1	5.2 ± 1.1	0.49
eGFR (ml/min/1.73 m^2^)	54 ± 20	47 ± 21	<0.001	54 ± 20	44 ± 21	<0.001
Creatinine (μmol/L)	133 ± 46	154 ± 83	0.003	132 ± 48	172 ± 91	<0.001
Proteinuria (>0.5 g/24 h) (*n*, %)	74 (15)	65 (40)	<0.001	83 (16)	56 (55)	<0.001
hs-CRP (mg/L)	1.5 (0.6–3.7)	2.5 (1.0–7.6)	<0.001	1.6 (0.7–4.3)	2.1 (0.8–6.1)	0.09
**TREATMENT**						
ACE-inhibitors (*n*, %)	157 (33)	58 (36)	0.47	176 (33)	39 (38)	0.29
Bèta-blocker (*n*, %)	300 (63)	113 (70)	0.08	346 (64)	67 (66)	0.81
Calcium channel blockers (*n*, %)	117 (25)	39 (24)	0.95	128 (24)	28 (28)	0.44
Diuretic use (*n*, %)	189 (40)	72 (45)	0.26	209 (39)	52 (51)	0.02
Calcineurin inhibitor (*n*, %)	281 (59)	92 (57)	0.57	315 (59)	60 (59)	0.44
Sirolimus (*n*, %)	10 (2)	2 (1)	0.33	11 (2)	1 (1)	0.50
Prednisolon, mg/24 h (*n*, %)	468 (99)	161 (100)	0.47	532 (99)	101 (99)	0.53
MMF (*n*, %)	294 (62)	87 (54)	0.10	328 (61)	55 (54)	0.70
Azathioprine (*n*, %)	77 (16)	41 (26)	0.68	93 (17)	27 (27)	0.71
**DONOR**						
Donor age (years)	46 ± 18	43 ± 15	0.07	43 ± 15	42 ± 16	0.29
Male sex donor (*n*, %)	232 (49%)	90 (56%)	0.11	280 (53%)	45 (46%)	0.19
Deceased type donor (*n*, %)	298 (62%)	117 (73%)	0.02	341 (63%)	77 (75%)	0.02
**HLA MISMATCHES (*****n*****, %)**						
Class I						0.46
0 (*n*, %)	102 (22)	27 (17%)		103 (19%)	26 (25%)	
1 (*n*, %)	113 (24%)	31 (19%)		125 (23%)	21 (20%)	
2 (*n*, %)	169 (36%)	53 (33%)		191 (36%)	33 (32%)	
3 (*n*, %)	43 (9%)	20 (12%)		51 (10%)	12 (12%)	
4 (*n*, %)	21 (4%)	9 (6%)		27 (5%)	3 (3%)	
Class II						0.82
0 (*n*, %)	199 (42%)	60 (37%)		215 (40%)	46 (45%)	
1 (*n*, %)	198 (42%)	66 (41%)		226 (42%)	40 (39%)	
2 (*n*, %)	47 (10%)	14 (9%)		52 (10)	9 (9%)	

*Normally distributed data are presented as means ± standard deviation, skewed data as medians (interquartile range), and categorical data as number (percentage). P-values have been calculated by means of independent samples T-test, Mann-Whitney U-test, or Chi-square test. eGFR, estimated glomerular filtration rate; hs-CRP, high-sensitivity C-reactive protein; ACE, angiotensin-converting enzyme; MMF, mycofenolaat mofetil; HLA, human leukocyte antigens*.

* *time since transplantation at inclusion*.

### Urinary Properdin and Graft Failure

**Figure 3:**
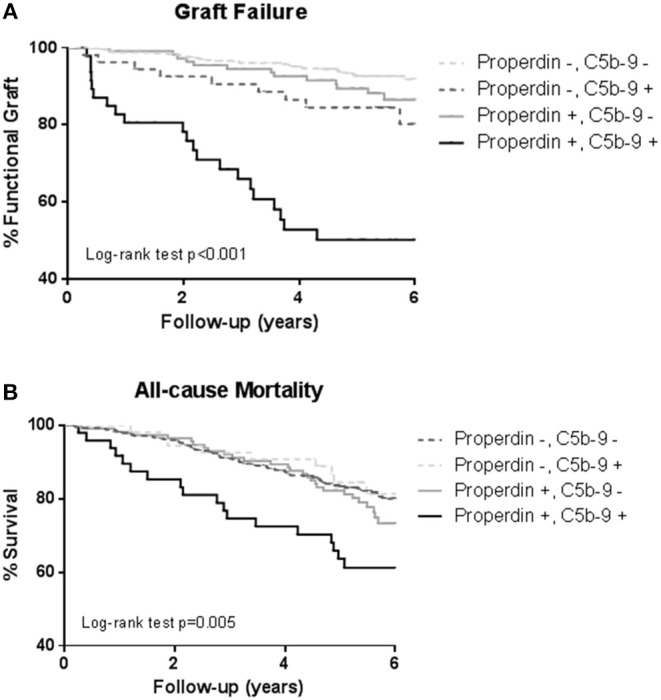
Kaplan-Meier analyses for percentage graft failure **(A)** and survival **(B)** according to no sC5b-9/no properdin, sC5b-9/no properdin, no sC5b-9/properdin, sC5b-9/properdin. Log-rank tests showed that the prevalence of graft failure and survival were significantly higher in the patients with urinary properdin and sC5b-9. Associations between survival and urinary properdin and sC5b-9 did not remain significant after adjustment for potential confounders.

During a median follow-up of 5.3 (4.5–6.0) years, 75 (12%) RTRs developed death-censored graft failure. As depicted in the Kaplan Meier curves shown in Figure 3, RTR with both detectable urinary properdin and sC5b-9 had the highest risk of developing graft failure (*P* < 0.001). RTR with urine in which either properdin or sC5b-9 was detectable, showed an intermediate risk with worse graft survival compared to RTR without detectable urinary properdin or sC5b-9 (Figure 3).

In unadjusted Cox regression analysis, detectable urinary properdin was significantly associated with development of death-censored graft failure (HR, 3.08; 95% CI 1.95–4.85; *P* < 0.001), in patients with neither urinary properdin or sC5b-9 as the reference group. In multivariable analyses, detectable urinary properdin remained associated with development of graft failure (HR, 2.30; 95% CI 1.37–3.82; *P* < 0.001, Table 2), independent of adjustment for age, sex, primary renal disease, time since transplantation, eGFR, HLA mismatches, donor type, hs-CRP, systolic blood pressure, and smoking. However, the association between detectable properdin and graft failure became borderline significant after further adjustment for proteinuria (HR, 1.47; 95% CI 0.85–2.54; *P* = 0.05).

**Table 2:** Association of detectable urinary properdin and detectable urinary sC5b-9 with graft failure in renal transplant recipients.

**Model**	**Detectable properdin**		**Detectable sC5b-9**		**Both properdin and sC5b-9**	
	**HR (95% CI)**	***P*-value**	**HR (95% CI)**	***P*-value**	**HR (95% CI)**	***P*-value**
Univariate	3.08 (1.95–4.85)	<0.001	4.17 (2.63–6.63)	<0.001	7.13 (4.30–11.83)	<0.001
Model 1	2.35 (1.44–3.82)	0.001	3.03 (1.86–4.96)	<0.001	8.04 (4.74–13.63)	<0.001
Model 2	2.27 (1.38–3.73)	0.001	2.99 (1.83–4.89)	<0.001	7.63 (4.46–13.10)	<0.001
Model 3	2.30 (1.37–3.82)	<0.001	3.09 (1.87–5.11)	<0.001	6.75 (3.79–12.02)	<0.001
Model 4	1.47 (0.85–2.54)	0.05	2.16 (1.30–3.61)	0.003	3.12 (1.69–5.77)	<0.001

***Model 1**, adjustment for age, sex, primary renal disease, time since transplantation at inclusion, eGFR, HLA mismatches, and donor type; **model 2**, model 1 + adjustment for hs-CRP; **model 3**, model 2 + adjustment for systolic blood pressure, and smoking; **model 4**, model 3 + adjustment for proteinuria*.

*Reference group defined as patients with neither urinary properdin or C5b-9, with a hazard ratio of 1.0*.

When we assessed the association between properdin as a continuous variable and graft failure, findings were similar. After adjustment for potential confounders, urinary properdin as a continuous variable was significantly associated with graft failure (HR, 1.25; 95% CI 1.10–1.42; *P* < 0.001) (Table 3). After adjustment for proteinuria, the association of properdin as a continuous variable with graft failure remained significant (HR, 1.12; 95% CI 1.02–1.38; *P* = 0.008). The optimal cut-off (Youden index) of urinary properdin for prediction of graft failure was 2.35 ng/mL. At this cut-off value, there was a sensitivity of 59% and a specificity of 79% for prediction of graft failure.

**Table 3:** Association of continuous natural log transformed urinary properdin and urinary sC5b-9 with graft failure in renal transplant recipients.

**Model**	**Ln properdin**		**Ln sC5b-9**	
	**HR (95% CI)**	***P*-value**	**HR (95% CI)**	***P*-value**
Univariate	1.36 (1.21–1.52)	<0.001	1.76 (1.51–2.06)	<0.001
Model 1	1.26 (1.11–1.43)	<0.001	1.61 (1.35–1.91)	<0.001
Model 2	1.25 (1.10–1.42)	0.001	1.61 (1.36–1.92)	<0.001
Model 3	1.25 (1.10–1.42)	0.001	1.63 (1.36–1.96)	<0.001
Model 4	1.12 (1.02–1.28)	0.008	1.34 (1.10–1.63)	0.004

***Model 1**, adjustment for age, sex, primary renal disease, time since transplantation at inclusion, eGFR, HLA mismatches, and donor type; **model 2**, model 1 + adjustment for hs-CRP; **model 3**, model 2 + adjustment for systolic blood pressure, and smoking; **model 4**, model 3 + adjustment for proteinuria*.

### Urinary sC5b-9 and Graft Failure

In unadjusted analysis, detectable urinary sC5b-9 was significantly associated with development of death-censored graft failure (HR, 4.17; 95% CI 2.63–6.63; *P* < 0.001). In multivariable analyses, detectable sC5b-9 remained associated with the development of graft failure (HR, 3.09; 95% CI 1.87–5.11; *P* < 0.001), independent of age, sex, primary renal disease, time since transplantation, eGFR, HLA mismatches, donor type, hs-CRP, systolic blood pressure, and smoking (Table 2). The association between detectable sC5b-9 and graft failure also remained after further adjustment for proteinuria (HR, 2.16; 95% CI 1.30–3.61; *P* = 0.003).

When we assessed the association between sC5b-9 as a continuous variable and graft failure, findings were similar. sC5b-9 as a continuous variable was associated with risk of developing graft failure in the unadjusted analysis and in multivariable analyses, after adjustment for potential confounders, including proteinuria (HR, 1.34; 95% CI 1.10–1.63; *P* = 0.004) (Table 3). The optimal cut-off (Youden index) of urinary sC5b-9 for prediction of graft failure was 2.88 ng/mL, there was a sensitivity of 48% and a specificity of 91% for prediction of graft failure.

### Mediation Analyses

Since properdin is involved in sC5b-9 complex formation via the alternative complement pathway (9), we aimed to assess whether the association between properdin and graft failure was mediated by sC5b-9. In mediation analyses, sC5b-9 was found to be a significant mediator of the association between properdin and graft failure, 31% of the association between properdin and graft failure was explained by sC5b-9, the *P* value for indirect effect is <0.05 (Table 4).

**Table 4:** Mediation analyses of the impact of sC5b-9 on the association between properdin and graft failure.

**Potential mediator**	**Outcome**	Effect (path)^*^	Multivariable model^**^	
			**Coefficient (95% CI)^†^**	Proportion mediated^***^
**C5b-9**	**Graft failure**	Indirect effect (*ab* path)	0.08 (0.04;0.13)	31%
		Total effect (*ab* + *c*' path)	0.26 (0.13;0.37)	
		Unstandardized total effect^‡^	0.22 (0.07;0.38)	

*Adjusted for age, sex, primary renal disease, time since transplantation, and hs-CRP*.

* *The coefficients of the indirect ab path and the total ab + c' path are standardized for the standard deviations of the potential mediators and outcomes*.

** *All coefficients are adjusted for age, sex, eGFR, time since transplantation at inclusion, primary renal disease, donor type and proteinuria*.

*** *The size of the significant mediated effect is calculated as the standardized indirect effect divided by the standardized total effect multiplied by 100*.

† *95% CIs for the indirect and total effects were bias-corrected confidence intervals after running 2,000 bootstrap samples*.

‡ *Odds ratios for risk of outcomes can be calculated by taking the exponent of the unstandardized total effect*.

### Properdin, sC5b-9, and Mortality

In an unadjusted Cox regression analysis, detectable urinary properdin was significantly associated with an increased risk of mortality (HR, 1.58; 95% CI 1.11–2.25; *P* = 0.01), whereas detectable urinary sC5b-9 was not significantly associated with increased risk of mortality(HR, 1.39; 95% CI 0.92–2.11; *P* = 0.12). After adjustment for potential confounders, the association between properdin and mortality was abrogated, and the association between sC5b-9 and mortality remained non-significant (Supplementary Table 1).

In unadjusted Cox regression analyses, both urinary properdin and sC5b-9 as continuous variables were significantly associated with an increased risk of mortality (HR, 1.16; 95% CI 1.05–1.27; *P* = 0.003 and HR, 1.26; 95% CI 1.08–1.48; *P* = 0.004, respectively). However, after adjustment for potential confounders, the associations between properdin and mortality, and between sC5b-9 and mortality were lost (Supplementary Table 2).

## Discussion

In this study, we show that the alternative pathway complement factor properdin and the terminal sC5b-9 complex are detectable in the urine of patients after kidney transplantation and are independently associated with chronic allograft failure. To our knowledge, this is the first report indicating urinary alternative complement pathway involvement in chronic renal allograft failure, independent of potential cofounders including eGFR and proteinuria.

It has long been recognized that patients with high-grade proteinuria are more likely to develop chronic renal failure than patients without proteinuria (23, 24). Urinary proteins elicit pro-inflammatory and pro-fibrotic effects that directly contribute to chronic tubulo-interstitial damage. Additionally, among multiple other pathways complement activation may be an important component leading to fibrogenesis in the kidney. In physiological conditions complement components are not filtered through the glomerular barrier, however complement components are present in the urine of patients with non-selective proteinuria (25, 26). Properdin positively regulates the AP of the complement system and is also a pattern recognition molecule for C3b that subsequently stabilizes the C3bBb complex and thus contributes to C5b-9 formation (27–30). Tubular epithelial cells are especially susceptible to the effects of C5b-9 formation because they lack the membrane-bound complement regulators on the apical cell surface (31). Recently, urinary complement measurements and their clinical value are of increasing interest in transplant medicine. Schröppel et al. showed not long ago the importance of anaphylatoxins C3a and C5a in donor urine and their association with delayed graft function (32), and van Essen et al. recently reviewed the detection of complement biomarkers in urine to monitor local injury in renal diseases, including properdin (33).

In this study, we have shown a potential role of urinary properdin and sC5b-9 in the pathogenesis of chronic allograft failure. Our data show that graft survival is reduced in patients in whom properdin is present in the urine together with sC5b-9. Remarkably, in patients without overt proteinuria, we identified that properdin, sC5b-9 or both properdin and sC5b-9, were also associated with a worse graft survival. More importantly, not only the presence of properdin and sC5b-9 was significantly associated with graft failure, but also properdin and sC5b-9 were robustly associated with graft survival when analyzed as continuous parameters, pointing toward a dose-dependent effect. There are several possible explanations for this association. Properdin is the only known complement protein that is not produced in the liver, but synthesized by various other cell types like monocytes, primary T cells, granulocytes, and endothelial cells (34–38). Therefore, it is possible that locally produced properdin and/or filtered properdin with other filtered small complement components, causes intratubular C5b-9 activation leading to progressive renal disease without manifest proteinuria, defined as proteinuria >0.5 g/24 h (39, 40). In kidney transplant patients it is generally believed that small amounts of proteinuria, defined as <0.5 g/24 h, are harmless (41, 42). Only persistent proteinuria, >0.5 g/24 h for at least 3–6 months is considered significant according to American Society of Transplantation guidelines, and low-grade proteinuria is often referred to as “subclinical” (43). However, low grade proteinuria may be less harmless than originally described. Halimi et al. showed a dose-dependent effect in transplant patients with low grade proteinuria (<0.5 g/24 h) in whom each 0.1 g/24 h difference in proteinuria increased the risk of graft loss by 25% (44). In line with our findings in transplanted patients, Siezenga et al. showed an association between urinary properdin and worse renal function in patients with diabetic nephropathy or glomerular disease. Furthermore, the association of urinary properdin with urinary sC5b-9 was independent of the degree of proteinuria (17).

The fact that adjustment for proteinuria > 0.5 g/24 h did not materially alter the prospective association in the prospective analysis of continuous properdin and sC5b-9 measurements, is supportive of our hypothesis that alternative pathway complement activation might be one of the driving forces of chronic graft failure. More importantly, after correction for other well-known predictors of graft failure, like HLA mismatches and donor type, the association remains. In mediation analysis, we showed that the association between properdin and graft failure was mediated to a considerable extent by urinary sC5b-9.

**Figure 4:**
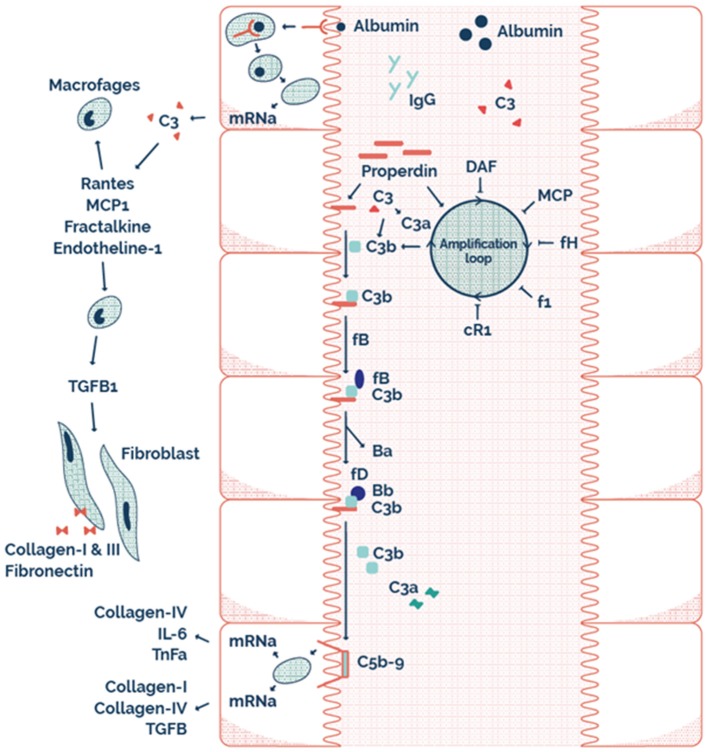
The possible mechanism on a tubular level illustrating tubular alternative pathway complement activation via properdin as pattern recognition molecule.

This may explain why RTR with properdin alone, or sC5b-9 alone in the urine have a better allograft survival compared to both properdin and sC5b9 in the urine. Therefore, it seems that AP complement activation plays an important role in the loss of allograft function of RTR. The possible mechanism of this effect at a tubular level is illustrated by the scheme presented in Figure 4. Interestingly, urinary properdin was more frequently detected in females and urinary sC5b-9 was more frequently detected in males. We can only speculate on the causes of these differences. Innate immune function may vary between males and females (45), however a limited number of studies have investigated the influence of sex on the complement system (46–49). Properdin is encoded on the short arm of the X chromosome, and together with hormonal differences between males and females this could be explanations of the sexual differences in properdin (50, 51). However, in a healthy Caucasian population, Gaya da Costa et al. recently found decreased serum properdin and serum C9 in healthy human females compared to males (49). In contrast, animal studies have shown that female mice have a similar serum complement cascade functionality at the level of C3 activation compared to male mice, but a strongly reduced level of serum C9, leading to an inability of female mice to promote inflammation through C5b-9 (46).

Multiple therapeutic modalities to inhibit complement pathway intervention are currently being developed. Our study points toward the potential for complement inhibition at the tubular level in proteinuric patients, which may improve long term outcome in patients with chronic allograft nephropathy.

The main strength of our study is that it comprises a large prospective cohort of stable RTR, in which several renal parameters as well as both urinary properdin and sC5b-9 were measured. In addition, end-point evaluation was complete in all participants despite the long follow-up period. We acknowledge several limitations of the study. First, no gold standard exists for the definition of urinary properdin and sC5b-9. In our study, we defined the detectability of properdin and sC5b-9 as urinary properdin and urinary sC5b-9. Second, complement activation may only be partially reflected by urinary properdin and sC5b-9 excretion, since the excretion may be altered by tubular complement binding and fixation. Third, possible residual confounding in this study cannot be excluded due to the observational status of this single center study. Furthermore, we do not have data on the presence of donor specific antibodies or protocol biopsies in this cohort. Thus, we cannot differentiate between general effects of glomerular filtration of complement products and a specific contribution of alloantibody mediated complement activation. Unfortunately our prospective cohort contained too few events of graft failure to perform analysis for the underlying cause of graft loss and their relation to urinary complement.

We identified that the presence of urinary properdin and sC5b-9 is independently associated with increased risk of late graft failure in RTR, compared to RTR without urinary properdin and sC5b-9. This suggests that urinary properdin and sC5b-9 can serve as useful biomarkers of immunological injury and kidney allograft deterioration. Importantly, urinary properdin and sC5b-9 was associated with graft failure independently of eGFR and significant proteinuria. We suggest that an important part of proteinuria mediated toxicity, is caused by the presence of complement in the primary urine and subsequent activation at the tubular surface. Further studies are needed to unravel the exact interplay between urinary properdin, sC5b-9 and the development of fibrosis, and moreover the potential for therapeutic interventions.

## Data Availability Statement

The datasets generated for this study are available on request to the corresponding author.

## Ethics Statement

The study was approved by the University Medical Center Groningen institutional review board (METc 2008/186), adheres to the Declarations of Helsinki and Istanbul and has NCT02811835 as ClinicalTrials.gov identifier. The patients/participants provided their written informed consent to participate in this study.

## Author Contributions

RL was involved in study design, carrying out assays, interpreting data, statistical analysis, creating tables and figures, and writing of the manuscript. ME was involved in interpreting data, statistical analysis, figure and table design, and manuscript editing. MA was involved in carrying out assays and interpreting data. MD, MS, RP, JB, and J-SS were all involved in interpreting data and manuscript editing. SB and SPB were involved in study design, interpreting data, statistical analysis, and manuscript editing.

### Conflict of Interest

The authors declare that the research was conducted in the absence of any commercial or financial relationships that could be construed as a potential conflict of interest.
