# Supplementary material for: Urinary Properdin and sC5b-9 Are Independently Associated With Increased Risk for Graft Failure in Renal Transplant Recipients
Source: Front Immunol. 2019 Oct 24;10:2511. doi: 10.3389/fimmu.2019.02511 (PMC6830301; doi:10.3389/fimmu.2019.02511)
Supplement: Supplementary file 1 [file Data_Sheet_1.PDF]

Online Supplementary Methods & Material

Urinary Properdin and sC5b-9 are Independently Associated with Increased Risk for Graft Failure in Renal Transplant Recipients

Rosa G.M. Lammerts[1], Michele F. Eisenga[1], Mohamed Alyami[1], Mohamed R. Daha[1], Marc A. Seelen[1], Robert A. Pol [2], Jacob van den Born[1], Jan-Stefan Sanders[1], Stephan J.L. Bakker[1], Stefan P. Berger[1], on behalf of the COMBAT Consortium

1 *Department of Nephrology, University Medical Center Groningen, University of Groningen, Netherlands*

2 *Department of Surgery, University Medical Center Groningen, University of Groningen, Netherlands*

Correspondence and request for reprints to:

Rosa G.M. Lammerts, M.D.

Department of Nephrology

University Medical Center Groningen

Hanzeplein 1, Z2.18, 9713 GZ Groningen

Email: r.g.m.lammerts@umcg.nl

Supplementary Figure 1. Association between urinary properdin and proteinuria excretion in the RTR. A restricted cubic spline is generated based on linear regression analyses. Knots are placed on 10th, 50th, and 90th percentile of proteinuria. Blue line represents coefficient, and pink band represents 95% confidence interval.

Supplementary Figure 2. Association between urinary soluble C5b-9 and proteinuria excretion in the RTR. A restricted cubic spline is generated based on linear regression analyses. Knots are placed on 10th, 50th, and 90th percentile of proteinuria. Blue line represents coefficient, and pink band represents 95% confidence interval.

Supplementary Table 1. Associations between detectable properdin and detectable sC5b-9 with all cause mortality in renal transplant recipients

	**Detectable properdin**		**Detectable sC5b-9**		**Both Properdin and sC5b-9**	
**Model**	**HR (95% CI)**	***P*-value**	**HR (95% CI)**	***P*-value**	**HR (95% CI)**	***P*-value**
Univariate	1.58 (1.11-2.25)	0.01	1.39 (0.92-2.11)	0.12	2.11 (1.28-3.46)	0.003
Model 1	1.43 (0.98-2.09)	0.07	1.06 (0.71-1.72)	0.66	1.87 (1.13-3.11)	0.02
Model 2	1.37 (0.93-2.00)	0.11	1.05 (0.67-1.64)	0.83	1.70 (1.01-2.86)	0.05
Model 3	1.30 (0.88-1.91)	0.19	1.00 (0.64-1.59)	0.99	1.20 (0.68-2.11)	0.53
Model 4	1.13 (0.75-1.70)	0.57	0.85 (0.53-1.33)	0.50	0.93 (0.52-1.66)	0.80

Model 1: adjustment for age, sex, primary renal disease, time since transplantation at inclusion, eGFR, HLA mismatches and donor type; **model 2** = model 1 + adjustment for hs-CRP, **model 3** = model 2 + adjustment for systolic blood pressure, and smoking, **model 4** = model 3 + adjustment for proteinuria.

Reference group defined as patients with neither urinary properdin or sC5b-9, with a hazard ratio of 1.0.

Supplementary Table 2. Associations between urinary properdin and sC5b-9 as continuous variables with all cause mortality in RTR

	Ln properdin		Ln sC5b-9	
**Model**	**HR (95% CI)**	***P*-value**	**HR (95% CI)**	***P*-value**
Univariate	1.16 (1.05-1.27)	0.003	1.26 (1.08-1.48)	0.004
Model 1	1.12 (1.01-1.24)	0.03	1.16 (0.97-1.38)	0.11
Model 2	1.10 (1.00-1.22)	0.06	1.14 (0.95-1.37)	0.15
Model 3	1.09 (0.99-1.21)	0.08	1.10 (0.91-1.33)	0.31
Model 4	1.06 (0.96-1.18)	0.27	1.01 (0.83-1.23)	0.92

Model 1: adjustment for age, sex, primary renal disease, and time since transplantation at inclusion, eGFR, HLA mismatches and donor type; **model 2** = model 1 + adjustment for hs-CRP, **model 3**= model 2 + adjustment for systolic blood pressure, and smoking, **model 4** = model 3 + adjustment for proteinuria
