# Supplementary figures and images for: Urinary Properdin and sC5b-9 Are Independently Associated With Increased Risk for Graft Failure in Renal Transplant Recipients
Source: Front Immunol. 2019 Oct 24;10:2511. doi: 10.3389/fimmu.2019.02511 (PMC6830301; doi:10.3389/fimmu.2019.02511)

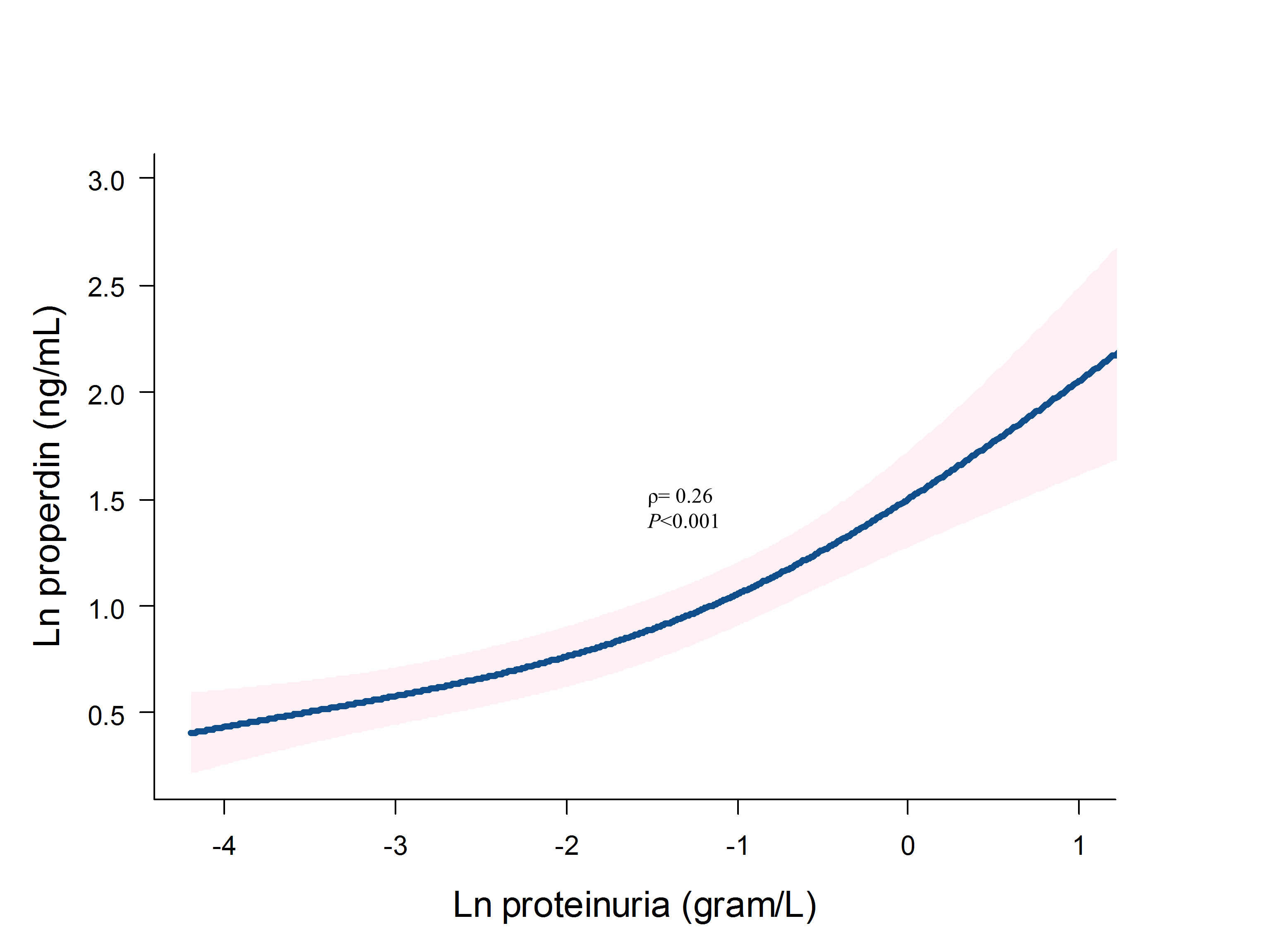

Supplement: Supplementary file 2 [file Image_1.tif]

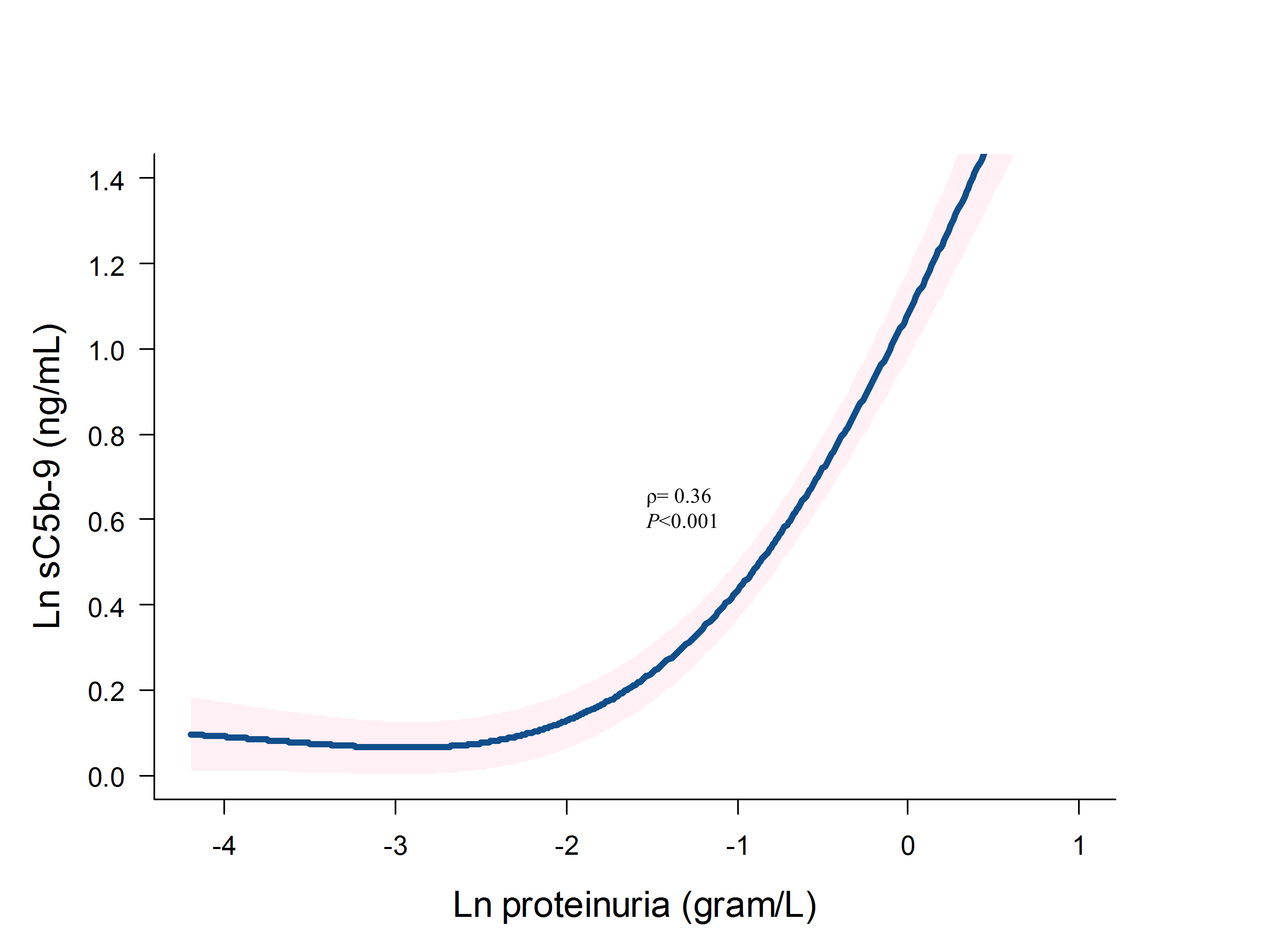

Supplement: Supplementary file 3 [file Image_2.tif]
